# Significance of abnormal 53BP1 expression as a novel molecular pathologic parameter of follicular-shaped B-cell lymphoid lesions in human digestive tract

**DOI:** 10.1038/s41598-021-82867-0

**Published:** 2021-02-04

**Authors:** Thi My Hanh Luong, Katsuya Matsuda, Daisuke Niino, Hirokazu Kurohama, Masahiro Ito, Masahiro Nakashima

**Affiliations:** 1 Department of Tumor and Diagnostic Pathology, Atomic Bomb Disease Institute, Nagasaki University Graduate School of Biomedical Sciences, 1-12-4 Sakamoto, Nagasaki, 852-8523 Japan; 2 Department of Pathology, Local Incorporated Administrative Agency Sasebo City General Hospital, Sasebo, Japan; 3 Department of Pathology, National Hospital Organization Nagasaki Medical Center, Omura, Japan

**Keywords:** Cancer, Molecular biology, Biomarkers, Gastroenterology, Oncology, Pathogenesis

## Abstract

The digestive tract is a common site of extranodal malignant lymphomas (MLs) and benign lymphoid lesions (BLs). TP53-binding protein 1 (53BP1) expression has been widely investigated in class switch recombination but rarely in human lymphoid tissues with respect to tumorigenesis. We previously reported that immunofluorescence (IF) analysis of 53BP1 nuclear foci (NF), reflecting DNA double strand breaks, is useful for estimating genomic instability in different tumor types. In this study, we evaluated the potential of IF-based analysis of 53BP1 expression in differentiating MLs from BLs. We examined 231 biopsied tissue samples of primary MLs and BLs in the digestive tract. The 53BP1 immunoreactivity pattern was determined by multicolor IF. Compared to BLs, MLs showed a high frequency of abnormal 53BP1 expression (*p* < 0.0001). Statistically, abnormal 53BP1 expression is an effective test for distinguishing follicular lymphomas from BLs (specificity 98.6%, sensitivity 86.8%) and for distinguishing small B-cell lymphomas from BLs (specificity 98.3%, sensitivity 77.6%). Furthermore, a high frequency of abnormal 53BP1 expression was associated with “high-risk” MALT lymphomas, which exhibited t(11;18)(q21;21) (*p* = 0.0145). Collectively, these results suggest that IF-based analysis of 53BP1 expression in biopsy samples is a promising technique for diagnosing MLs in the digestive system.

## Introduction

The gastrointestinal tract is the most common site of extranodal non-Hodgkin lymphoma^1–3^ as well as a common site of reactive lymphoid hyperplasia because of acquired immune response resulting from continuous exposure to several foreign bodies, including food, alcohol, chemicals, microbiomes, and other inflammatory substances^4–6^. Biopsy-based differential diagnosis between malignant lymphomas (MLs) and benign lymphoid lesions (BLs) is difficult because of the limited amount of sample and similar histological features, such as follicular/nodular shapes and/or small cell size, as well as low mitotic activity^7^.

DNA double strand breaks (DSBs) occur as part of normal B- and T- lymphocyte development during the assembly of variable (V), diversity (D), and joining (J) gene segments into the V-region coding exons via V(D)J recombination^8^. Furthermore, class switch recombination, a mechanism that changes a B cell’s production from one type of immunoglobulin to another,^9^ and secondary V(D)J rearrangement in mature CD4^+^ T-cells also involve the generation of DSBs^10^. Improper repair of DSBs in these processes may induce chromosomal instability, causing tumorigenesis.

The tumor suppressor p53-binding protein 1 (53BP1), a DNA damage response (DDR) molecule possessing a BRCA1 C-terminal domain^11,12^, rapidly forms nuclear foci (NF) at the site of DNA DSBs in response to ionizing radiation^13–15^. Therefore, we previously hypothesized that the presence of 53BP1 NF indicates endogenous DNA DSBs and DDR activation in individual cells, and that the 53BP1 expression pattern may be useful for estimating genomic instability, which is a known hallmark of malignancy, during thyroid, bladder, uterine cervix, and skin carcinogenesis^16–21^. The 53BP1 immunofluorescent pattern was classified into five types: i) stable (none or weak nuclear staining); ii) low DDR (one or two discrete NF); iii) high DDR (HDDR; three or more discrete NF); iv) large focus (LF; discrete NF ≥ 1 µm); and v) diffuse (intense and heterogenous staining)^17^. We previously showed that both the HDDR and LF patterns were closely associated with increased cervical carcinogenesis upon human papilloma virus infection^17^, and both LF and diffuse patterns were significantly associated with high-grade urothelial carcinoma with chromosomal instability and poor prognosis^18^. In the present study, we investigated the 53BP1 expression pattern as an indicator of genome instability, in lymphoid tissues of the GI tract. We observed considerable differences in the pattern of 53BP1 expression between MLs and BLs, and found that frequently abnormal 53BP1 NF expression was associated with “high-risk” mucosa-associated lymphoid tissue (MALT) lymphomas exhibiting chromosomal translocation. To the best of our knowledge, this is the first study to reveal differences in the 53BP1 expression pattern during lymphoid tumorigenesis.

## Results

### Profiles of evaluated cases

Of the 107 cases of primary ML analyzed, 97 cases (90.7%) were the B-cell type and 10 cases (9.3%) were the T-cell type (Table 1). These T-cell lymphomas consisted of 6 cases of adult T-cell leukemia/lymphoma, one case of enteropathy-associated, and 3 cases of other types. To clarify the association between the type of 53BP1 expression and nuclear grade of T-cell lymphoma, we classified these cases into two groups based on their cell-size, such as a small or large cell size. The sex ratio (M/F) was 1.8, and the median age (interquartile range: IQR) at diagnosis was 68 (60.5–75) and 70 (62.5–73.8) years in women and men, respectively. The 124 BL cases evaluated in this study included 37 cases (29.8%) of chronic gastritis, 42 cases (33.9%) of non-specific enteritis/colitis or ulcers, 2 cases (1.6%) of ischemic colitis, 4 cases (3.2%) of Crohn’s disease, 17 cases (13.7%) of ulcerative colitis, 15 cases (12.1%) of adenocarcinoma, 1 case (0.8%) of neuroendocrine tumor, 4 cases (3.2%) of adenoma, and 2 cases (1.6%) of hyperplastic polyp. For BL cases, the sex ratio (M/F) was 1.4 and median age (IQR) at diagnosis was 59 (41–72.3) and 57.5 (46–68) years in women and men, respectively.

**Table 1:** Profiles of evaluated cases.

		n	M/F	Age (years)^a^	Sites				Abnormal type 53BP1 expression (%)^a^
					Stomach	Small intestine	Colon	Others	
Benign	GC^b^	25	2.1	58.5 (40–71)	9	5	10	1 (IC)	15.6 (9.3–19.9)
	PF	48	1.4	58 (47–72)	20	8	20	0	20.8 (18.9–23.6)
	MM^b^	44	1.4	59 (40–74)	18	9	16	1 (IC)	23.9 (19–26.3)
	LA	28	1.3	56 (41–69)	13	4	10	1 (IC)	23.3 (18.8–27.7)
Malignant	MALT	19	1.4	60.5 (54.8–68.3)	19	0	0	0	19.3 (14.7–34.1)
	MCL	11	10	71.5 (66.8–72.5)	2	4	4	1 (SD)	44.5 (32–52.9)
	FL1	14	1.3	69 (53.5–73)	0	12	1	1 (IC)	52.1 (40.6–56)
	FL2	18	2.6	67 (61–76)	0	16	2	0	41.9 (35.2–51.5)
	FL3A	6	0.5	63 (61–67.5)	0	6	0	0	29.8 (26.4–33)
	DLBCL	29	1.6	73 (66–81)	18	6	3	2 (IC)	27.6 (20.3–34.8)
	TL^c^	10	2.3	72.5 (68–73)	4	6	0	0	29.9 (23.9–38.6)
	Small	5	1.5	70.5 (68–73)	3	2	0	0	29.9 (25.5–37.2)
	Large	5	4	66.5 (58.8–72.8)	1	4	0	0	30 (20.6–38.8)

*M/F* male/female, *SD* stomach and duodenum, *IC* ileocecum or Bauhin’s valve, *GC* germinal center, *PF* primary follicle, *MM* mantle-marginal zone, *LA* simple lymphoid accumulation, *MALT* mucosa-associated lymphoid tissue lymphoma, *MCL* mantle cell lymphoma, classical type, *FL 1, 2, 3A* follicular lymphoma grade 1, 2, 3A, *DLBCL* diffuse large B cell lymphoma, *TL* T cell lymphoma.

^a^Median (interquartile range).

^b^23 cases were evaluated for both GC and MM.

^c^TLs were subtyped by cell size: “small” consisted of *small* and *medium cells*, “large” consisted of *large cells* over than 70% (s*mall cells*: 1–2 × size of mature small lymphocyte, *large cells*: larger than 3 × size of mature small lymphocyte, *medium cells*: 2–3 × size of mature small lymphocyte).

### 53BP1 expression patterns in MLs

**Figure 1:**
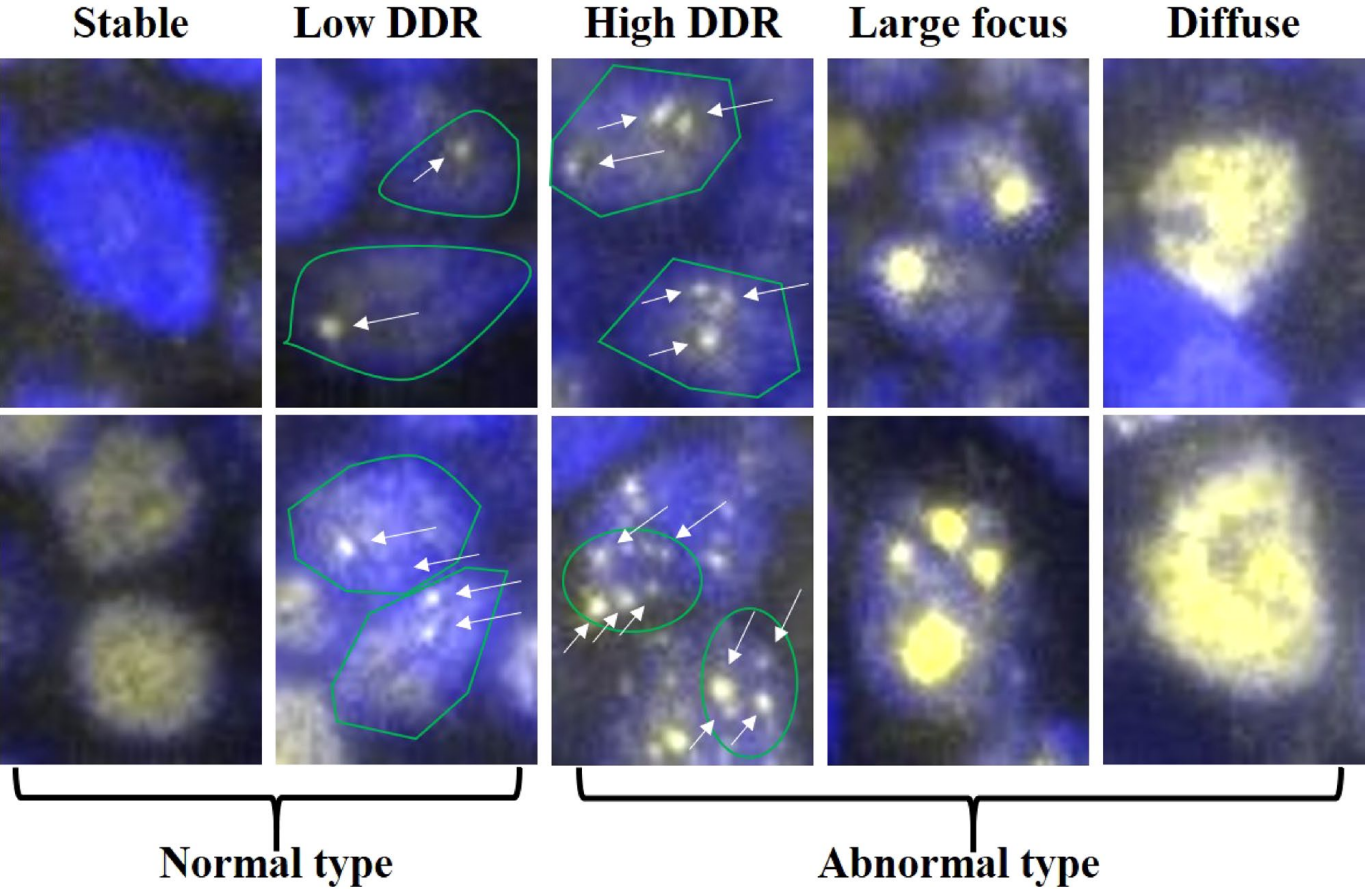
Types of 53BP1 expression patterns observed in lymphocytes by immunofluorescence. Stable type, no or weak staining; low DNA damage response (DDR), one or two nuclear foci (NF, indicated by arrows) with size < 1 µm; high DDR, three or more NF (indicated by arrows) with size < 1 µm; large focus, NF with size ≥ 1 µm; diffuse type, intense and heterogenous staining. Green line indicates the border of the nucleus. Images for 53BP1 expression were photographed with the Z-stack function of Biorevo BZ-X710 microscope (Keyence Japan, Osaka, Japan), and their signals were measured using the image analysis software provided with the Biorevo BZ-X710.

**Figure 2:**
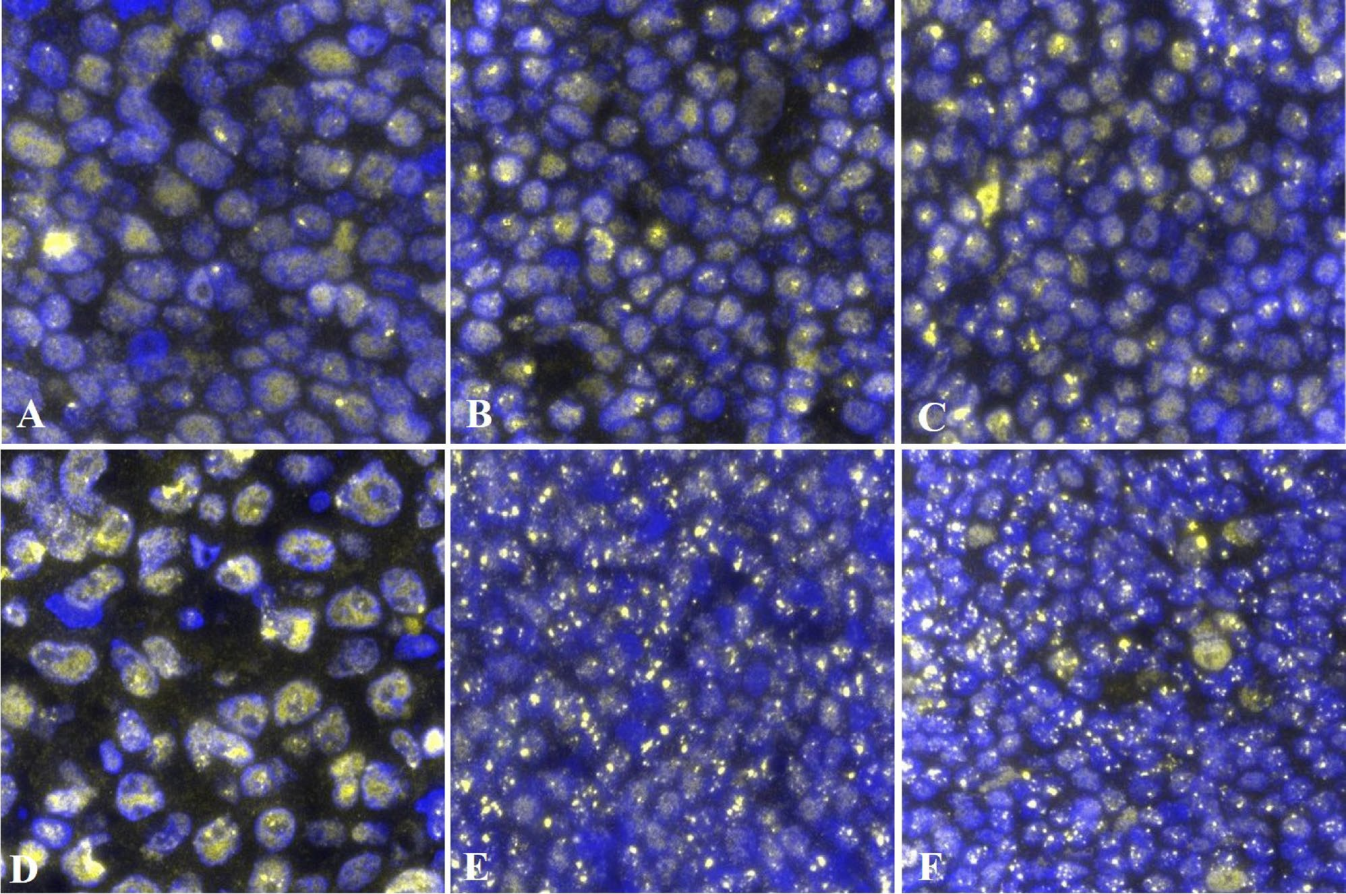
Representative images of 53BP1 expression in benign and malignant lymphoid proliferative lesions. **(A)** Germinal center; **(B)** mantle-marginal zone; **(C)** primary follicle; **(D)** diffuse large B-cell lymphoma; **(E)** mantle cell lymphoma, classical type; and **(F)** follicular lymphoma grade 1. Numerous abnormal types of 53BP1 expression are shown in both **(E)** and **(F)**. The images were captured after identifying anatomical structures with BCl2 and FDC expression as shown in Supplementary Fig. 1. Images for 53BP1 expression were photographed with the Z-stack function of Biorevo BZ-X710 microscope (Keyence Japan, Osaka, Japan), and their signals were measured using the image analysis software provided with the Biorevo BZ-X710.

**Figure 3:**
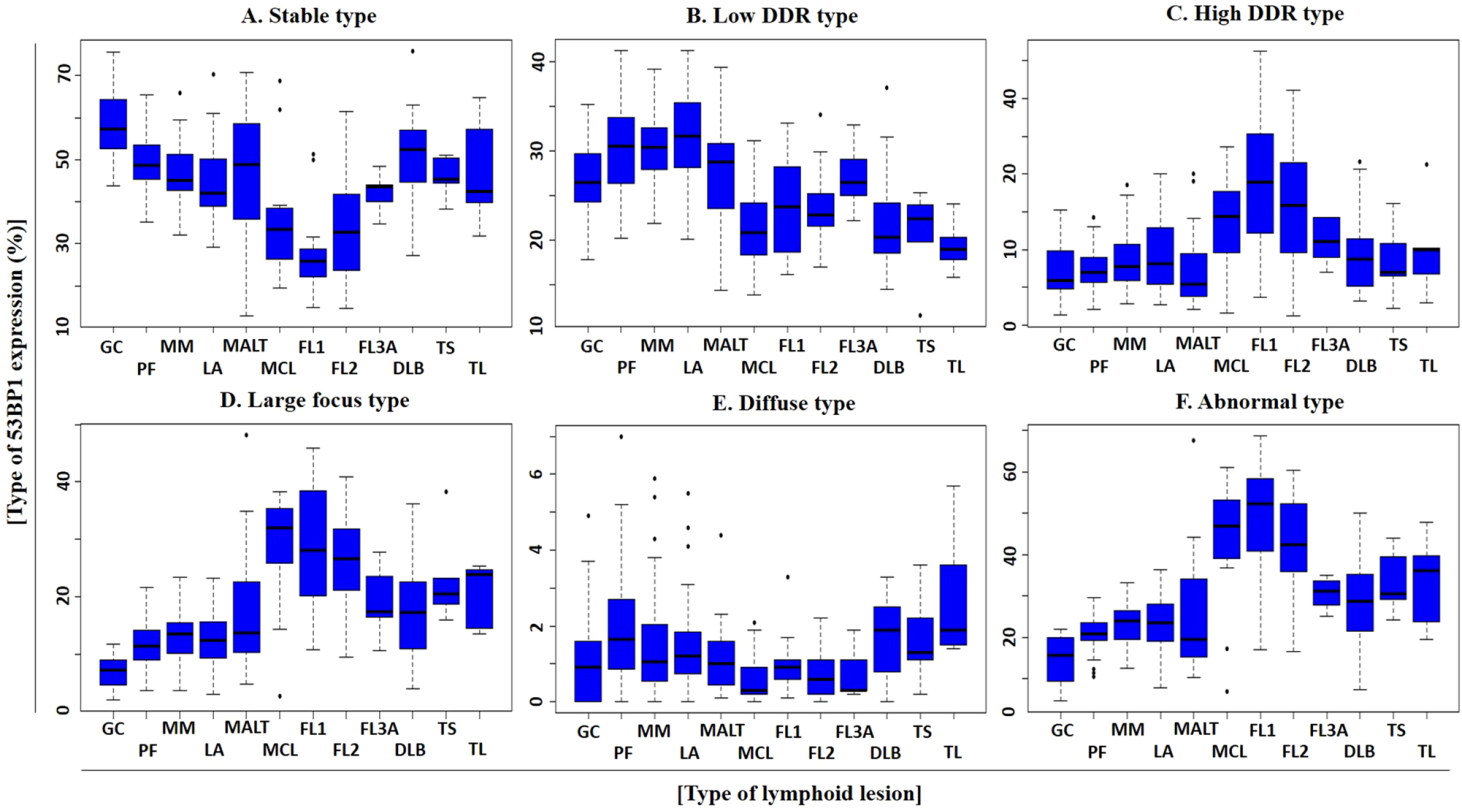
Schematic presentation of results for quantitative analysis of 53BP1 expression in various lymphoid proliferative lesions based on type of 53BP1 expression. Box plots showing frequencies (%) of **(A)** stable, **(B)** low DNA damage response (DDR), **(C)** high DDR, **(D)** large focus, **(E)** diffuse, and **(F)** abnormal types. *GC* germinal center, *PF* primary follicle, *MM* mantle-marginal zone, *LA* simple lymphoid accumulation, *MALT* mucosa associated lymphoid tissue lymphoma, *MCL* mantle cell lymphoma, classical type, *FL1, 2, 3A* follicular lymphoma grade 1, 2, 3A, *DLBCL* diffuse large B-cell lymphoma, *TS* T-cell lymphoma, small cell size, *TL* T-cell lymphoma, large cell size.

We first identified the typical 53BP1 expression patterns in BL and ML tissues and determined the distribution of each pattern in the lymphoid lesions**.** Stable and low DDR types are considered as normal, whereas high DDR, large focus, and diffuse patterns are considered as abnormal types (Fig. 1). The frequency of abnormal 53BP1 expression types in the lymphoid lesions is shown in Table 1. Most nuclei in the BLs [including germinal center (GC), primary follicle (PF), mantle-marginal zone (MM), simple lymphoid accumulation (LA)] were stable or the low DDR type, with a low frequency of the abnormal type, whereas several nuclei in the mantle cell lymphoma (MCL) and follicular lymphoma (FL) tissues showed abnormal expression patterns (Figs. 2 and 3). We also compared the abnormal type in different follicular lymphoid lesions (GC, PF, and FLs) and small B-cell type lymphoid lesions (PF, MM, LA, FLs, and MCL). Among the follicular lymphoid lesions, the frequency of abnormal 53BP1 expression types in FLs [including FL grade 1 (FL1), FL grade 2 (FL2) and FL grade 3A (FL3A)] was significantly higher than that in GC and PF (Table 2; Figs. 2 and 3). Among small B-cell lymphoid lesions, the frequency of abnormal type in both FLs and MCL was significantly higher than that in BLs, except for FL3A vs. LA (Table 3; Figs. 2 and 3). The frequency of the abnormal patterns in MLs decreased significantly with an increasing histological grade of FLs [FL1 > FL2 > FL3A > diffuse large B cell lymphoma (DLBCL; germinal center type)] (linear regression model, *p* < 0.0001; Table 1; Fig. 3). Interestingly, in a case of MCL showing both classical and aggressive (blastoid) types, abnormal expression of 53BP1 was observed in 52.6% of nuclei in the classical type but only 21.9% of nuclei in the blastoid variants (Supplementary Fig. 2). The frequency of abnormal expression in small T-cell lymphoma was significantly higher than that in both GC and PF (*p* = 0.0253 and 0.0389, respectively), but not in MM and LA. The frequency of the abnormal type in DLBCL was significantly higher than that in both GC and PF (*p* = 0.0004 and 0.0211, respectively) but not in MM and LA. No significant differences were observed in the abnormal type of 53BP1 expression between BLs and MALT/large T-cell lymphoma (data not shown).

**Table 2:** *p*-value in comparisons of abnormal type 53BP1 expression among follicular-shape lymphoid lesions.

	PF	FL1	FL2	FL3A
GC	0.0002	< 0.0001	< 0.0001	0.0017
PF		< 0.0001	< 0.0001	0.0014
FL1			0.6117	0.0786
FL2				0.1152

*GC* germinal center, *PF* primary follicle, *FL 1, 2, 3A* follicular lymphoma grade 1, 2, 3A.

**Table 3:** *p*-value in comparisons of abnormal type 53BP1 expression among small cell lymphoid lesions.

	MM	LA	MCL	FL1	FL2	FL3A^a^
PF	0.2701	0.5270	0.0117	< 0.0001	< 0.0001	0.0028
MM		0.9997	0.0125	< 0.0001	< 0.0001	0.0299
LA			0.0245	0.0003	0.0002	0.1647
MCL				0.9797	0.9999	0.3454
FL1					0.7886	0.1395
FL2						0.1984

*PF* primary follicle, *MM* mantle-marginal zone, *LA* simple lymphoid accumulation, *MCL* mantle cell lymphoma, *FL 1, 2, 3A* follicular lymphoma grade 1, 2, 3A.

^a^These cases mainly consisted of small cells but large cells (centroblasts and immunoblasts) less than 10%

**Figure 4:**
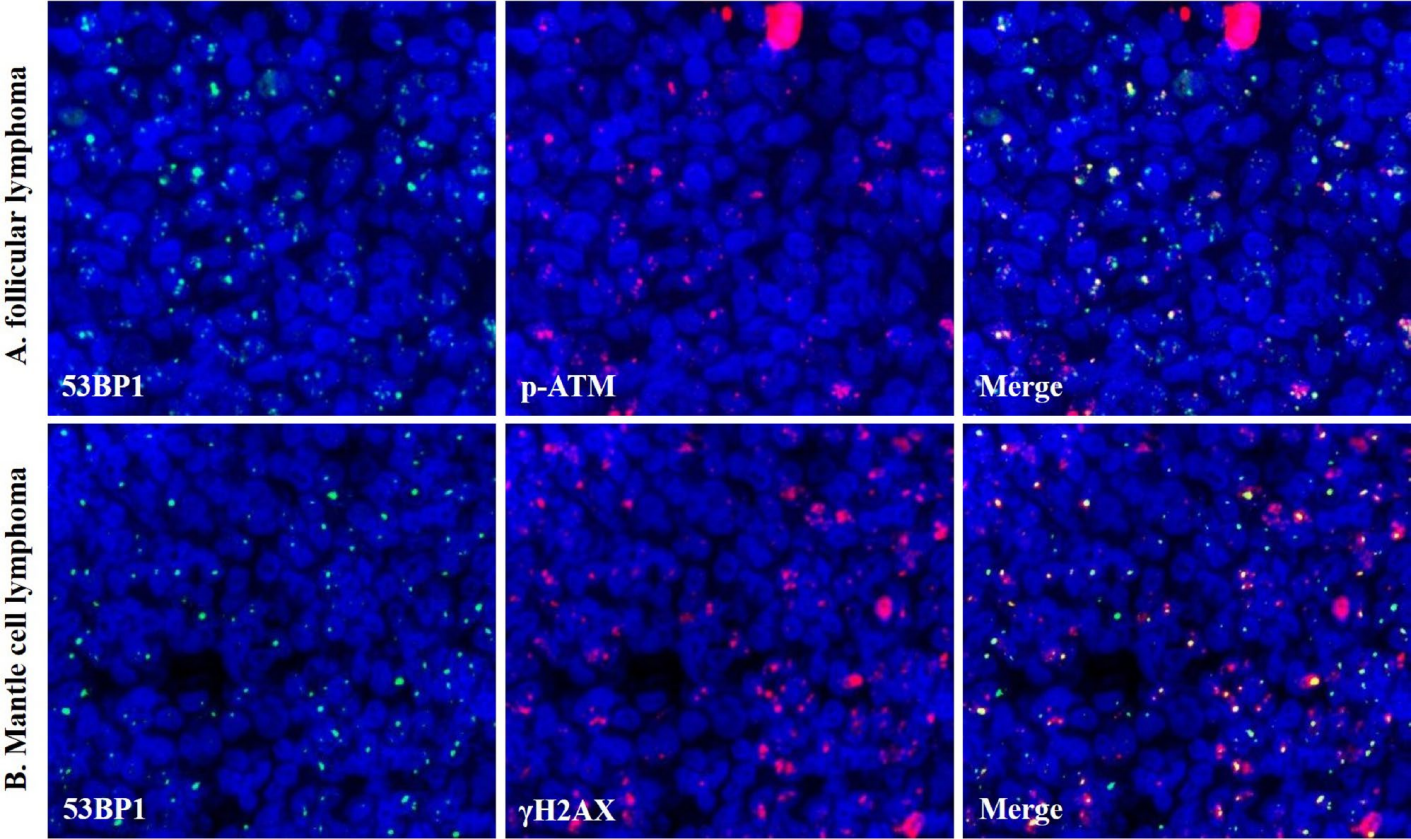
Co-localization of 53BP1and DDR-related molecules. Co-localization of 53BP1 (green) and p-ATM (orange) in follicular lymphoma grade 2 **(A)**, and 53BP1 (green) and γH2AX (orange) in mantle cell lymphoma, classical type **(B)** by dual-color immunofluorescence analyses. The images were photographed with the Z-stack function of Biorevo BZ-X710 microscope (Keyence Japan, Osaka, Japan), and their signals were analysed using the image analysis software provided with the Biorevo BZ-X710.

Dual-color IF analysis revealed frequent co-localization of 53BP1 expression and other DDR-related molecules, γH2AX (γ histone-2AX) and p-ATM (phosphorylated ataxia telangiectasia mutated) NF, in the MLs (Fig. 4).

### Relationship between abnormal type of 53BP1 expression and Ki-67 labeling index (LI) in FLs

We investigated the relationship between Ki-67 LI and grades of FLs and Ki-67 LI and abnormal type of 53BP1 expression. As expected, Ki-67 LI [median (IQR range)] was higher in FL3A [13.8% (12.5–16.8)] than in FL2 [5.2% (4.3–10.1)] and FL1 [5.0% (2.4–7.1)] (*p* = 0.0194 and 0.0021, respectively). In contrast, the frequency of an abnormal type of 53BP1 expression was decreased in higher grade FLs, whereas there was no significant correlation between the frequency of an abnormal type and Ki-67 LI (Spearman correlation coefficient = − 0.26, *p* = 0.1208).

### Abnormal 53BP1 expression indicates of B-cell MLs

**Figure 5:**
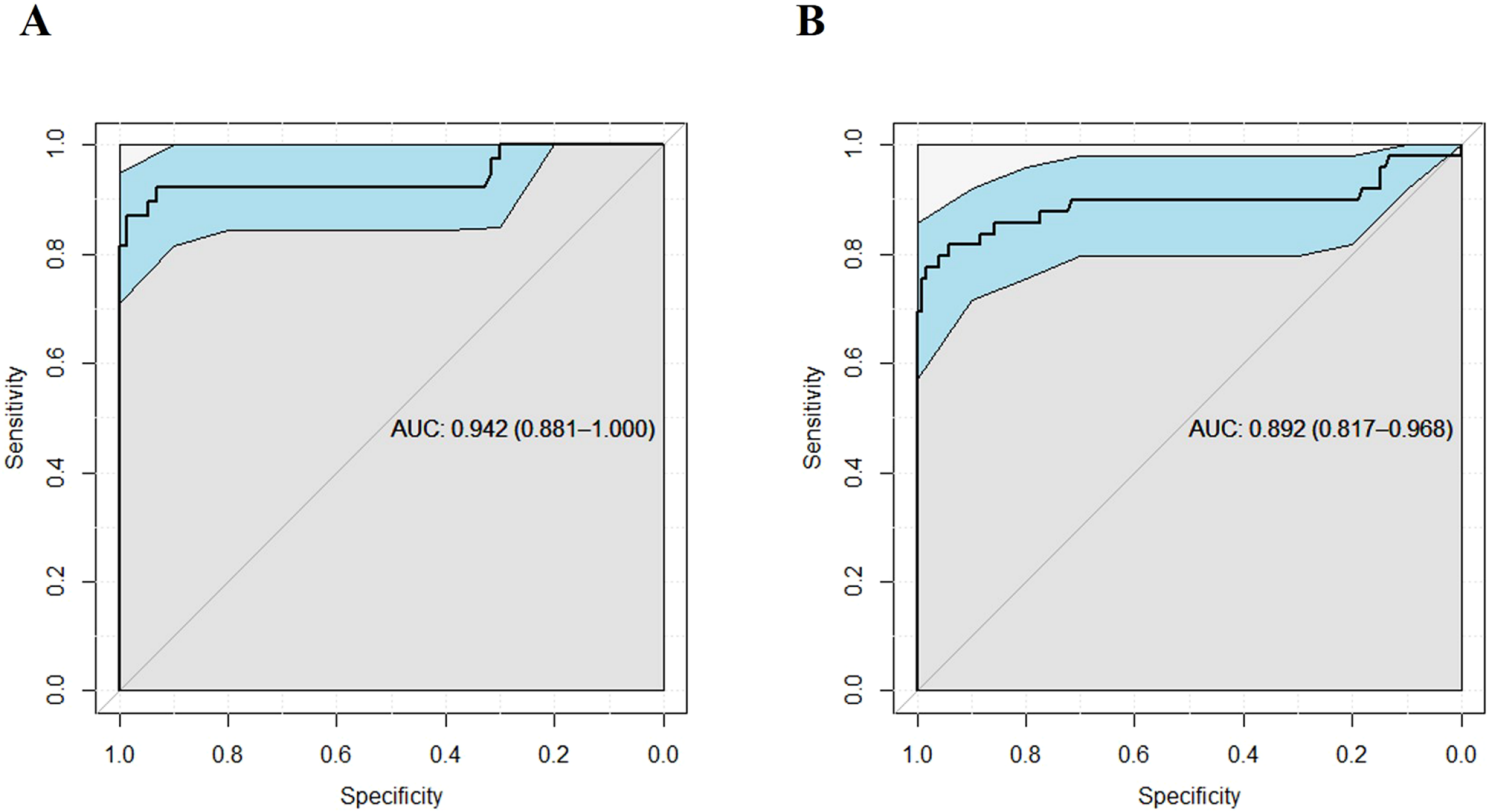
ROC curves for detecting malignant lymphomas using the ratio of abnormal types of 53BP1 expression by immunofluorescence. **(A)** ROC curve for follicular lymphoid proliferative lesions, such as follicular lymphomas vs. germinal center (n = 111). Area under the curve is 0.942 (95% confidence interval, 0.861–1.000, *p* < 0.0001, logistic regression model). **(B)** ROC curve for small cell lymphoid proliferative lesions, such as mantle cell lymphoma vs. mantle-marginal zone (n = 169). Area under the curve is 0.892 (95% confidence interval, 0.817–0.968, *p* < 0.0001, logistic regression model).

Next, we focused on the ratio of nuclei with an abnormal type of 53BP1 expression as an indicator to distinguish primary B-cell MLs from BLs in the biopsy specimens and assessed the receiver operating characteristic (ROC) curve. Among follicular lymphoid lesions (GC, PF, and FLs), the area under the curve (AUC) value was 0.942 [95% confidence interval, 0.881–1.000], suggesting that FLs were reliably detected (Fig. 5A). When 27.2% was considered as the cut-off value (Youden’s index, 0.85) for the FL diagnosis, the sensitivity and specificity were 86.8% and 98.6%, respectively. Furthermore, among small cell lymphoid lesions (PF, MM, LA, FLs, and MCL), the AUC value was 0.892 (95% confidence interval, 0.817–0.968), suggesting reliable detection of MLs (Fig. 5B). When 33.6% was considered as the cut-off value (Youden’s index, 0.76) for the small-cell ML diagnosis, the sensitivity and specificity were 77.6% and 98.3%, respectively. In addition, among the “large B-cell” group including GC and DLBCL, the AUC value was 0.859 (95% confidence interval, 0.755–0.962). When 22.9% was considered as the cut-off value (Youden’s index, 0.72) for the large B-cell ML diagnosis, the sensitivity and specificity were 72.4% and 100%, respectively.

### Association between abnormal 53BP1 expression, and chromosomal aberrations and response to therapy in MALT lymphomas

**Figure 6:**
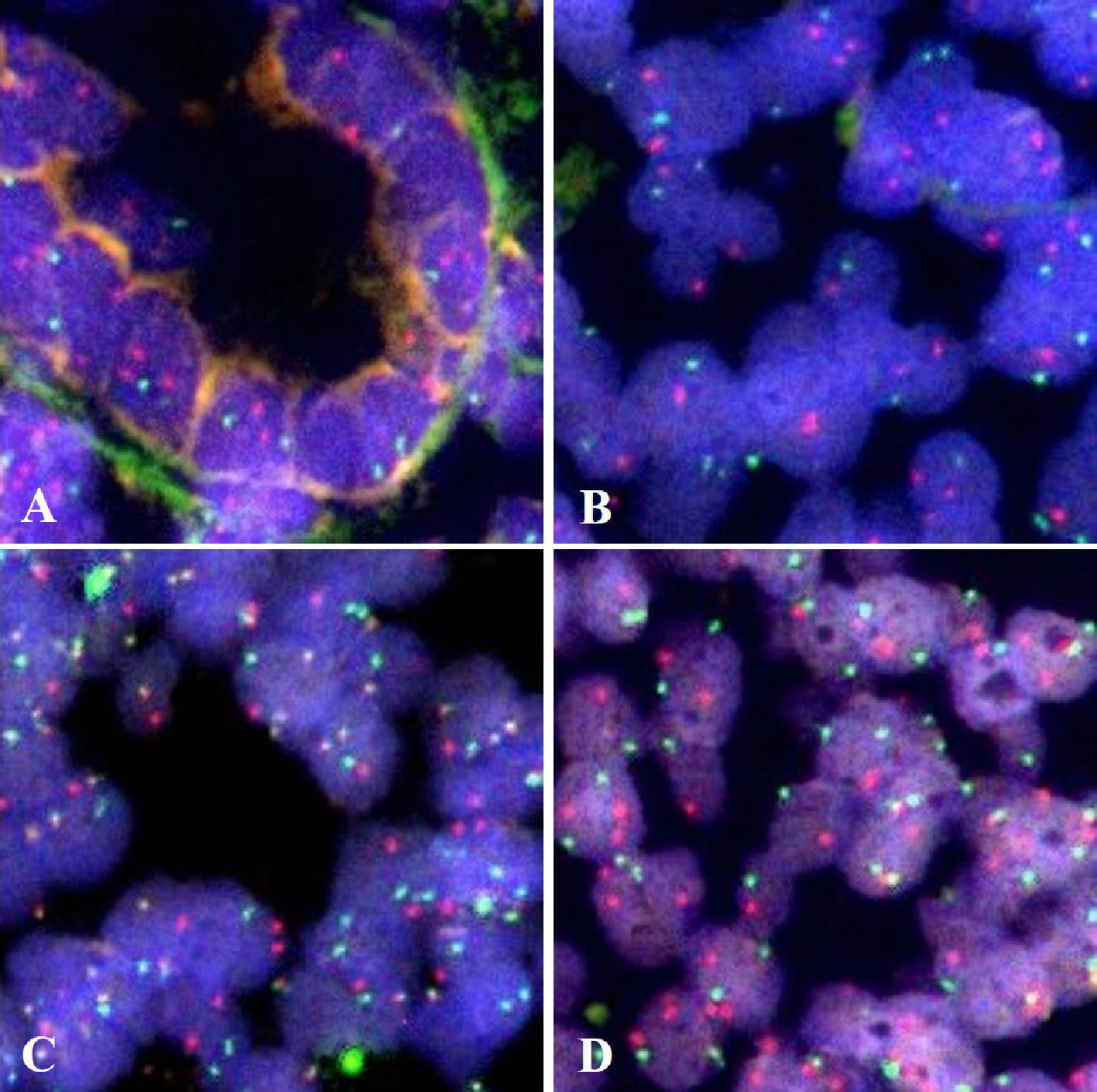
Dual-color interphase FISH analysis to detect chromosomal aberrations of 11q22 (BRIC3, green) and 18q21 (MALT1, orange) in MALT lymphomas. **(A)** Wild type signals in normal gastric gland (A) and in a case of MALT lymphoma with a low frequency of abnormal 53BP1 expression (Case No. 8 in Table 4) **(B)**. Translocation t(11;18)(q21;q21) signals (yellow) in a case of MALT lymphoma with a high frequency of abnormal type (Case No. 3 in Table 4) **(C)**. Trisomy of 18q21 (three orange and two green signals) in a case of MALT lymphoma with a high frequency of abnormal type (Case No. 2 in Table 4) **(D)**. The images were photographed with the Z-stack function of Biorevo BZ-X710 microscope (Keyence Japan, Osaka, Japan), and their signals were analysed using the image analysis software provided with the Biorevo BZ-X710.

The results of molecular analyses obtained by fluorescence in situ hybridization (FISH), response to *Helicobacter pylori* eradication and radiation therapy, and follow-up data in MALT lymphomas, are summarized in Table 4. Six (31.6%) of the 19 MALT lymphoma cases showed a high frequency of the abnormal type of 53BP1 expression (more than 33.6%, which is the cut-off value for diagnosing small B-cell type MLs). Interestingly, 5 (83.3%) of these cases showed t(11;18)(q21;q21), and two showed trisomy of 18q21 (Fig. 6, Table 4). Another case that exhibited a high frequency (44.2%) of the abnormal type also presented a low frequency (less than 10%) of 18q21 trisomy. Among the 13 cases with a low frequency of the abnormal type, one case exhibited t(11;18)(q21;q21), 9 cases were wild type, and 3 cases showed no detectable signals in FISH analysis. Statistical analysis revealed a significant association between the abnormal type of 53BP1 expression and chromosomal aberrations (*p* = 0.0145, Wilcoxon exact test). Therapy and follow-up data were available for 14 (73.7%) of 19 MALT lymphoma cases, including 5 and 9 of the high and low abnormal type cases, respectively. Among them, spontaneous regression or response to therapies was confirmed in 2 (40%) of five cases with the high abnormal type and in 7 (77.8%) of nine cases with low abnormal type. Furthermore, among the seven cases with eradication, all three responders were the low abnormal type.

**Table 4:** Results of molecular analyses and response to therapy in MALT lymphomas.

No	Age (years)	Sex	Abnormal type of 53BP1 expression (%)	t(11;18)(q21;q21)	Trisomy of 18q21	*H. pylori* eradication	Radiation	Response to therapy or regression
1	69	M	67.7	+	+	+	−	−
2	69	M	34.1	+	+	+	−	−
3	74	F	42.9	+	−	−	−	−
4	56	F	36.9	+	−	−	+	+
5	68	M	34.1	+	−	−	+	+
6	47	F	19.1	+	−	+	−	+
7	59	M	44.2	−	+ /−^a^	ND	ND	ND
8	87	F	26.5	−	−	−	−	+
9	59	M	26.5	−	−	ND	ND	ND
10	66	M	19.4	−	−	−	+	+
11	42	F	18	−	−	+	−	−
12	58	M	17.2	−	−	+	−	DT
13	74	F	15	−	−	+	−	+
14	50	M	13.9	−	−	ND	ND	ND
15	59	M	11.4	−	−	+	−	−
16	62	M	10.3	−	−	−	+	+
17	51	F	13.7	NS	NS	+	−	+
18	67	F	15.2	NS	NS	−	+	+
19	65	M	24.1	NS	NS	ND	ND	ND

*NS* no signals, *ND* no data, *DT* during therapy.

^a^Nuclei with trisomy < 10%

## Discussion

53BP1 expression in animal lymphoid tissues or cultured human lymphocytes has been widely investigated in terms of class switch recombination^22–27^; however, studies of 53BP1 expression in human lymphoid tissues with respect to tumorigenesis are limited. In this study, we determined the difference between the frequency of abnormal 53BP1 expression in biopsied ML and BL specimens from the gastrointestinal tract.

Using irradiated rat thyroid glands, we previously demonstrated that the presence of DDR-type 53BP1 immunoreactivity was concordant with the induction of DNA DSBs in irradiated follicular cells^28^. Furthermore, our previous studies involving dual-color IF analysis revealed frequent co-localization of 53BP1 and γH2AX NF in human thyroid follicular tumors and irradiated rat thyroid glands, suggesting endogenous activation of the DDR pathways in tumor cells as a hallmark of genome instability^28^. Thus, we hypothesized that 53BP1 expression is a useful tool for estimating the level of genome instability and malignant potential of human tumors. In this study, 53BP1 was found to co-localize with other DDR molecules, such as γH2AX and p-ATM NF, in human MLs. DNA DSBs do not only occur in MLs^29^, but also in the development of B- and T-cells^8–10^. Although 53BP1 NF may appear in both benign lymphocytes and ML cells, the frequency of abnormal 53BP1 expression in MLs was significantly higher than that in BLs, indicating an association of altered DDR machinery with lymphoid tumorigenesis as well as other carcinogenesis. We previously showed that an abnormal type of 53BP1 NF was closely associated with increased carcinogenesis of several organs^16–18,21^; for instance, both LF and diffuse patterns were significantly associated with high-grade urothelial carcinoma with chromosomal instability and poor prognosis^18^. This suggests a more universal association with carcinogenesis rather than V(D)J recombination-mediated DSB generation which is specific to lymphoid cells.

This study also revealed a stepwise decrease in the frequency of abnormal type 53BP1 expression with ML progression [FL1 > FL2 > FL3A > DLBCL (germinal center type)]. Additionally, we observed a 2.4-fold higher frequency of abnormal type 53BP1 expression in the area of classical type compared to in the area of more aggressive blastoid variant in one MCL case. These findings suggest the involvement of a different type of DDR machinery in more aggressive MLs. Although the precise mechanism underlying this phenomenon remains poorly understood, our result is consistent with that of a previous study showing that the DDR pathway is impaired in more aggressive tumors, enabling them to escape DNA repair and apoptosis induction^30^. Therefore, high-grade MLs may lack the ability to respond to DNA damage, thereby showing increased genome instability and more aggressive phenotypes.

Genomic profiling studies have identified the key pathways activated in the pathogenesis of mature B-cell neoplasms, such as B-cell receptor (BCR) signaling, NOTCH signaling, Toll-like receptor (TLR), phosphoinositide 3 kinase (PI3K)/AKT/mTOR signaling, and mitogen activated kinase (RAS/MAPK/ERK) pathways^31^. BCR antigen binding induces the recruitment of Src family kinases and, subsequently, activating PI3K-related kinases, which are the major transducers of the DNA damage signals, executing functional outcomes in the DDR^32^. NOTCH signaling provokes an increase in S-phase and directly regulates the DDR by inactivating ATM, resulting in a decreased level of residual 53BP1 NF, which is crucial for the proliferation of T-cell leukemias^33^. TLR initiates intracellular signaling by engaging various cytoplasmic adaptors that acts as a signal transducer, including MYD88. MYD88 mutations results in uncontrolled formation of the MYD88/IL-1 receptor-associated kinase (IRAK) complex, recruitment of the tumor necrosis factor receptor-associated factor (TRAF) 6 which is an E3 ubiquitin ligase, and constitutive phosphorylation of NF-κB signaling activation, which can activate MAPK pathway and respond to radiation-induced DNA damage^34–36^. Thus, activation of these pathways can influence 53BP1 expression to enhance lymphomagenesis through impaired DDR.

**Figure 7:**
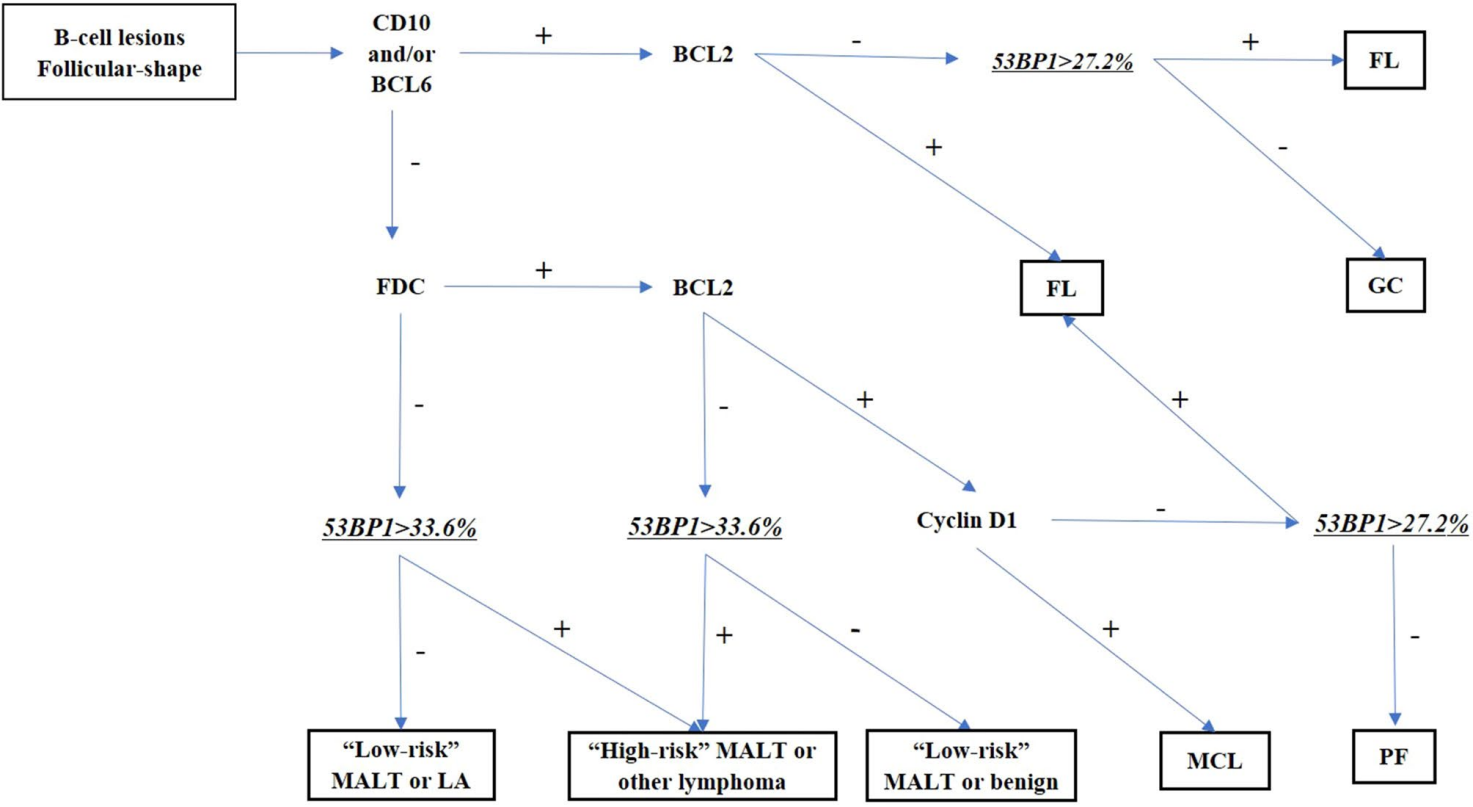
Proposed flow chart to diagnose follicular-shape B-cell lymphoid lesions in biopsy samples from the digestive tract by a combination of usual immunohistochemistry and immunofluorescence of 53BP1. *GC* germinal center, *PF* primary follicle, *LA* simple lymphoid accumulation, *FL* follicular lymphoma, *MCL* mantle cell lymphoma, *MALT* mucosa-associated lymphoid tissue lymphoma. “High-risk” MALT: showing t(11;18)(q21;q21) and insensitivity to *H. pylori* eradication therapy. “Low-risk” MALT: showing no chromosomal translocation and sensitivity to *H. pylori* eradication therapy.

Histopathological diagnosis of MLs using biopsy samples from the gastrointestinal tract, particularly follicular lesions, is not always easy because of the small size of the cells and insufficient recognizable features^7,37^. Immunohistochemistry-based detection of CD10 and BCL2 co-expression is used to diagnose FLs in biopsy samples. However, 20% of FL1/2 and 83% of FL3 have been reported to be negative for CD10 expression^38^, and 8.8% of FLs have been reported as negative for BCL2 expression^39^. BCL6 is another marker used to diagnose FLs, but 30.5% of FLs do not show BCL6 expression^40^. Therefore, we investigated the potential of 53BP1 IF analysis as a useful technique for distinguishing FLs from BLs. The present study proposes a method for distinguishing follicular-shape B-cell lymphoid lesions, including FL, in biopsy samples from the gastrointestinal tract by a combination of usual immunohistochemistry and 53BP1 IF (Fig. 7). Our results indicate that IF-based analysis of 53BP1 expression is an outstanding diagnostic test for distinguishing FLs (AUC, 0.94; CI, 0.88–1; cut-off, 27.2%) and excellent diagnostic test for distinguishing small B-cell MLs from BLs (AUC, 0.89; CI, 0.82–0.97; cut-off, 33.6%), according to the previously reported classification^41^ (Fig. 5). However, the clinical application of this test has some limitations, such as the need for a fluorescence microscope and training to evaluate the staining results, which require an understanding of morphological features.

Although there was no significant difference between 53BP1 expression in MALT lymphoma and BLs, we found a correlation between the high frequency of the abnormal type (more than 33.6%, cut-off value to diagnose small B-cell MLs) and t(11;18)(q21;q21)/trisomy of 18q21 (*p* = 0.0145). The incidences of t(11;18)(q21;q21) and trisomy 18 in gastric MALT lymphoma have been reported as 6–26% and 6%, respectively^42^. Moreover, cases with t(11;18)(q21;q21) are typically insensitive to *H. pylori* eradication therapy, suggesting the prognostic significance of these cases as “higher-risk” as compared with those without^43–45^. We propose that a high level of abnormal type 53BP1 expression is a useful parameter for estimating chromosomal instability as well as the prognosis in gastric MALT lymphomas.

In summary, this retrospective study indicates that the frequency of abnormal type 53BP1 expression in lymphoid proliferative lesions in biopsy specimens from the gastrointestinal tract is an attractive diagnostic test to distinguish MLs from BLs. Furthermore, the logistic regression model with ROC curve analysis revealed that the proportion of cells showing an abnormal type of 53BP1 expression by IF analysis is a very effective diagnostic parameter for follicular lymphoid lesions (AUC, 0.94; CI, 0.88–1; cut-off, 27.2%) and for small cell lymphoid lesions (AUC, 0.89; CI, 0.82–0.97; cut-off, 33.6%). Furthermore, we found a stepwise decrease in the frequency of abnormal type with FL progression, suggesting the involvement of different DDR machinery impairments in aggressive feature of FLs. Additionally, abnormal types of 53BP1 expression appear to be associated with chromosomal instability, a parameter of prognosis in gastric MALT lymphoma. Thus, IF-based analysis of 53BP1 expression may be a useful tool for distinguishing MLs from BLs in biopsy samples from the gastrointestinal system. If it is technically possible to automate the quantification of 53BP1 NF by using a computational image analysis system, our method may be useful for practical diagnosis of follicular lymphoid lesions.

## Methods

### Subjects

We analyzed 107 cases of primary MLs in the gastrointestinal tract, diagnosed at hospitals (National Hospital Organization Nagasaki Medical Center, Nagasaki University Hospital, Isahaya General Hospital) in Nagasaki, Japan, between 2008 and 2019. A total 124 cases of inflammatory diseases or non-lymphoid tumors in the gastrointestinal tract were also examined as controls for BLs. All available samples were biopsied, formalin-fixed, and paraffin-embedded tissues histologically showing lymphoid accumulation. The final pathological diagnosis was verified by at least three experienced pathologists following the diagnostic criteria of WHO 2017.

This study was performed retrospectively and in accordance with the tenets of the Declaration of Helsinki. The Ethics Committee of Nagasaki University approved the study (approval date: June 20, 2019; #15062617-3). The need for informed consent was waived by the Ethics Committee of Nagasaki University. Methods were carried out in accordance with relevant guidelines and regulations. All patient profiles were anonymized by coding and collectively summarized with the obtained data as final dataset. Detailed information regarding this study is available on our website (http://www-sdc.med.nagasaki-u.ac.jp/ pathology/research/index.html). Patients were able to opt out of study by following the instructions provided on the faculty website.

### Multicolor IF analyses of 53BP1 expression

After deparaffinization and antigen retrieval by microwave treatment for 20 min in citrate buffer (pH 6.0), the tissue sections were pre-incubated with 10% normal goat serum. For triple-color IF, the tissues were incubated with polyclonal rabbit anti-53BP1 antibody (1:1000; Bethyl Laboratories, Montgomery, TX, USA), monoclonal mouse anti-human BCL-2 antibody (1:50; DakoCytomation, Glostrup, Denmark), and monoclonal mouse anti-human FDC (follicular dendric cell) antibody (CNA.42; 1:200; Life Technologies, Carlsbad, CA, USA). The samples were then incubated with Alexa Flour 532-conjugated goat anti-rabbit, Alexa Fluor 488-conjugated goat anti-mouse, and Alexa Fluor 594 F(abʹ)-conjugated goat anti-mouse antibodies (Molecular Probes, Eugene, OR, USA). For dual-color IF, the tissues were incubated with polyclonal rabbit anti-53BP1 antibody (1:1000) and monoclonal mouse anti-γ histone-2AX (γH2AX) antibody (1:1000; ab26350; Abcam, Cambridge, UK) or with monoclonal mouse anti-ataxia telangiectasia mutated (ATM; phospho S1981) antibody (1:500; ab36810; Abcam). The samples were then incubated with Alexa Fluor 488-conjugated goat anti-rabbit and Alexa Fluor 594 F(abʹ)-conjugated goat anti-mouse antibodies (Molecular Probes) as appropriate. All slides were mounted using Vectashield HardSet Mounting Medium containing DAPI (Vector Labs, Burlingame, CA, USA).

The stained sections were photographed at 1,000-fold magnification with at least 30 slices per field using the Z-stack function on a High Standard all-in-one fluorescence microscope (Biorevo BZ-X710; Keyence Japan, Osaka, Japan). This function enabled delineation of all 53BP1 foci throughout the nucleus. In BLs, triple-color IF images were captured after determining the anatomical structures (Supplementary Fig. 1), such as the germinal center (GC; FDC-positive and BCL2-negative), mantle-marginal zone (MM; BCL2-positive area surrounding GC), primary follicle (PF; FDC-positive and BCL2-positive), and simple lymphoid accumulation (LA; FDC-negative and variable BCL2 expression).

All signals for 53BP1 expression were measured using the image analysis software provided with the Biorevo BZ-X710 microscope. 53BP1 immunoreactivity was classified using the definitions as mentioned in the Introduction, according to our previous report^17^ (Fig. 1). The percentage of nuclei containing each type of expression was calculated for each case.

### Dual-color FISH analysis of MALT lymphoma

We detected the translocation t(11;18)(q21;q21) by dual-color FISH with Vysis LSI BIRC3/MALT1 probes (Abbott Japan Co. Ltd., Tokyo, Japan) following the manufacturer’s instructions. Briefly, after deparaffinization and microwave treatment in citrate buffer (pH 6.0), the tissue sections were predigested with 0.075% pepsin for 20 min at 37 °C, re-fixed in 4% paraformaldehyde for 5 min at 4 °C, and denatured in 70% formamide for 5 min at 73 °C. The tissue sections were incubated with denatured FISH probes, including SpectrumGreen-labeled BRIC3 and SpectrumOrange-labeled MALT1, at 37 °C for 16 h. The sections were washed in 50% formamide and 2 × standard saline citrate and counterstained with DAPI (Vector Labs). A minimum of 30 slices per field were visualized and photographed using the Z-stack function of Biorevo BZ-X710 (Keyence Japan). Non-overlapping nuclei of more than 150 cells with signals were evaluated per slide. The proportion of nuclei with one or two fusion signals in total nuclei showing at least one hybridization signal was calculated.

### Statistical analyses

Continuous variables were expressed as the median and IQR, and the groups were compared using Wilcoxon rank-sum test. A logistic regression model and ROC curve were used to evaluate the significance of 53BP1 expression using IF as a diagnostic test. An optimal cutoff value was chosen based on the highest Youden’s index. All statistical analyses were performed using the SAS software (version 9.4, SAS Institute Inc., Cary, NC, USA) with a statistical significance level of 0.05. The boxplots and ROC curves were created in R statistical language version 3.6.1 by using ggplots, dplyr, and pROC packages.

## Supplementary Information

Supplementary Figures.
