# Author Correction: Significance of abnormal 53BP1 expression as a novel molecular pathologic parameter of follicular-shaped B-cell lymphoid lesions in human digestive tract

**DOI:** 10.1038/s41598-021-91766-3

**Published:** 2021-06-09

**Authors:** Thi My Hanh Luong, Katsuya Matsuda, Daisuke Niino, Hirokazu Kurohama, Masahiro Ito, Masahiro Nakashima

**Affiliations:** 1 Department of Tumor and Diagnostic Pathology, Atomic Bomb Disease Institute, Nagasaki University Graduate School of Biomedical Sciences, 1‑12‑4 Sakamoto, Nagasaki, 852-8523 Japan; 2 Department of Pathology, Local Incorporated Administrative Agency Sasebo City General Hospital, Sasebo, Japan; 3 Department of Pathology, National Hospital Organization Nagasaki Medical Center, Omura, Japan

Correction to: *Scientific Reports* https://doi.org/10.1038/s41598-021-82867-0, published online 04 February 2021

The original version of this Article contained errors in the spelling of the author Thi My Hanh Luong, which was incorrectly given as Luong Thi My Hanh.

In addition, Figure 7 contained an error where “53BP1 > 27.2%” was missing from the flow chart.

These errors have now been corrected in the PDF and HTML versions of the Article and the Supplementary Information file that accompanies the Article.

