# Supplementary material for: Significance of abnormal 53BP1 expression as a novel molecular pathologic parameter of follicular-shaped B-cell lymphoid lesions in human digestive tract
Source: Sci Rep. 2021 Feb 4;11:3074. doi: 10.1038/s41598-021-82867-0 (PMC7862599; doi:10.1038/s41598-021-82867-0)

Significance of abnormal 53BP1 expression as a novel molecular pathologic parameter of follicular-shaped B-cell lymphoid lesions in human digestive tract

Thi My Hanh Luong,[1] Katsuya Matsuda,[1] Daisuke Niino,[2] Hirokazu Kurohama,[1] Masahiro Ito,[3] and Masahiro Nakashima[1]*

Supplementary figure legends

Supplementary figure 1. Dual-color immunofluorescence for follicular dendritic cell (FDC) and BCL2 expression to identify anatomical structures of reactive lymphoid lesions, such as germinal center (GC), mantle-marginal zone (MM), primary follicle (PF), and simple lymphoid accumulation (LA).

Supplementary figure 2. A case of mantle cell lymphoma (MCL) of the rectum showing both classical cell type and aggressive blastoid features. (A) Classical MCL; (B) Aggressive blastoid MCL with a high mitotic activity. Immunofluorescence analysis for abnormal 53BP1 expression pattern in (C) classical MCL and (D) blastoid MCL.

Supplementary figure 1

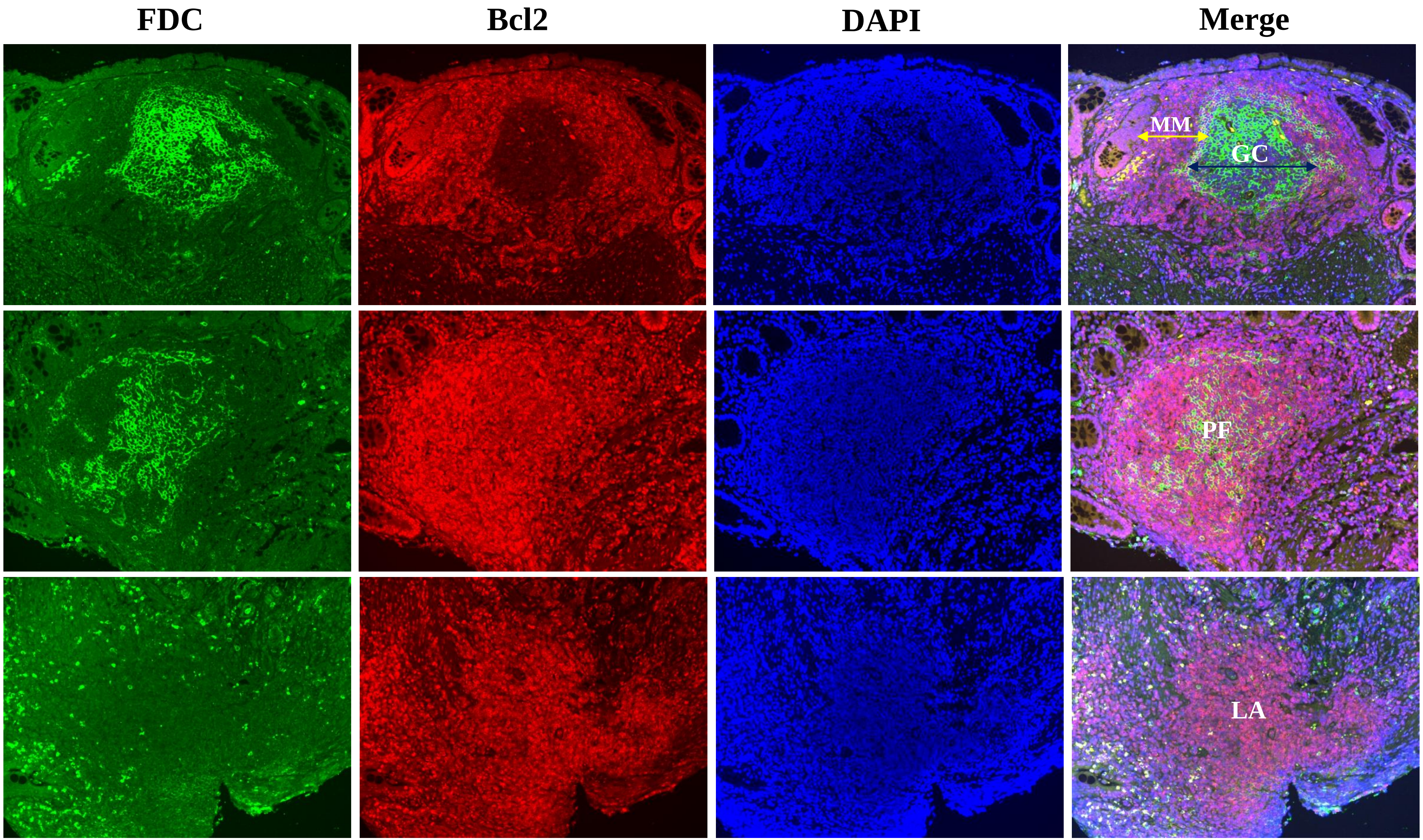

Supplementary figure 2

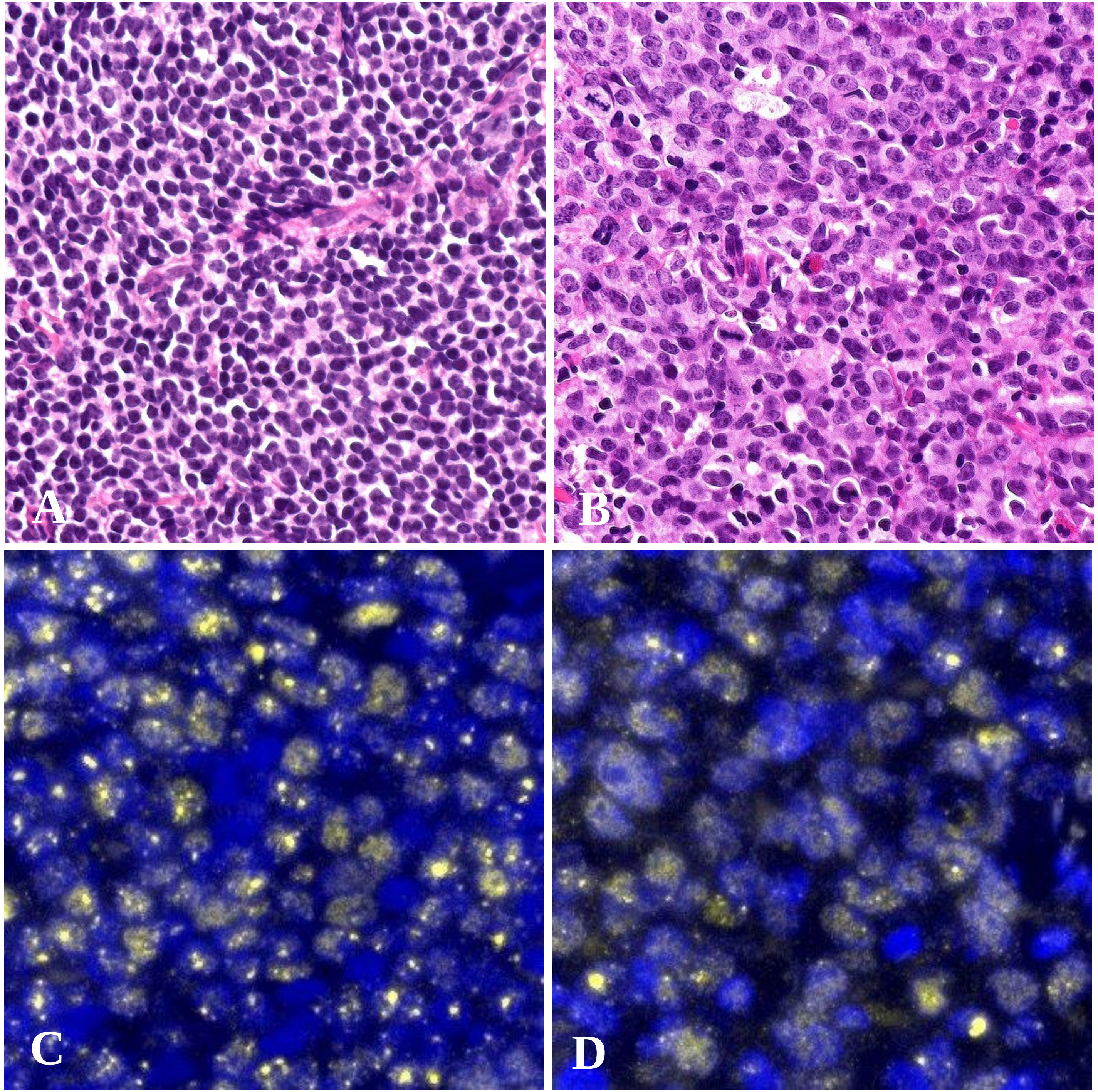

Supplement: Supplementary file 1 — Supplementary Figures. [file 41598_2021_82867_MOESM1_ESM.pdf]
